# Androgen receptor gain in circulating free DNA and splicing variant 7 in exosomes predict clinical outcome in CRPC patients treated with abiraterone and enzalutamide

**DOI:** 10.1038/s41391-020-00309-w

**Published:** 2021-01-26

**Authors:** M. Del Re, V. Conteduca, S. Crucitta, G. Gurioli, C. Casadei, G. Restante, G. Schepisi, C. Lolli, F. Cucchiara, R. Danesi, U. De Giorgi

**Affiliations:** 1 Unit of Clinical Pharmacology and Pharmacogenetics, Department of Clinical and Experimental Medicine, University Hospital of Pisa, Pisa, Italy; 2 Department of Medical Oncology, Istituto Scientifico Romagnolo per lo Studio e la Cura dei Tumori (IRST) IRCCS, Meldola, Italy

**Keywords:** Predictive markers, Cancer therapy

## Abstract

**Background:**

Androgen receptor (AR) signaling inhibitors represent the standard treatment in metastatic castration resistance prostate cancer (mCRPC) patients. However, some patients display a primary resistance, and several studies investigated the role of the AR as a predictive biomarker of response to treatment. This study is aimed to evaluate the role of AR in liquid biopsy to predict clinical outcome to AR signaling inhibitors in mCRPC patients.

**Methods:**

Six milliliters of plasma samples were collected before first-line treatment with abiraterone or enzalutamide. Circulating free DNA (cfDNA) and exosome-RNA were isolated for analysis of AR gain and AR splice variant 7 (AR-V7), respectively, by digital droplet PCR.

**Results:**

Eighty-four mCRPC patients received abiraterone (*n* = 40) or enzalutamide (*n* = 44) as first-line therapy. Twelve patients (14.3%) presented *AR* gain and 30 (35.7%) AR-V7+ at baseline. Median progression-free survival (PFS) and overall survival (OS) were significantly longer in AR-V7− vs AR-V7+ patients (24.3 vs 5.4 months, *p* < 0.0001; not reached vs 16.2 months, *p* = 0.0001, respectively). Patients carrying the *AR* gain had a median PFS of 4.8 vs 24.3 months for *AR* normal patients (*p* < 0.0001). Median OS was significantly longer in *AR* normal vs patients with *AR* gain (not reached vs 8.17 months, *p* < 0.0001). A significant correlation between AR-V7 and *AR* gain was observed (*r* = 0.28; *p* = 0.01). The AR gain/AR-V7 combined analysis confirmed a strong predictive effect for biomarkers combination vs patients without any AR aberration (PFS 3.8 vs 28 month, respectively; OS 6.1 vs not reached, respectively; *p* < 0.0001).

**Conclusions:**

The present study demonstrates that cfDNA and exosome-RNA are both a reliable source of AR variants and their combined detection in liquid biopsy predicts resistance to AR signaling inhibitors.

## Introduction

Prostate cancer is the most frequent cancer in men and is rated as the second cause of cancer deaths in the Western world [1]. The signaling of the androgen receptor (AR) axis plays an essential role in prostate cancer development and progression. Although a majority of patients are initially responsive to androgen deprivation therapy (ADT), most will eventually develop castration resistance prostate cancer (CRPC) [2]. Several findings showed that AR signaling persists during castration [3], leading to the introduction of AR-directed therapies for metastatic CRPC (mCRPC), such as abiraterone and enzalutamide, which significantly prolonged patients’ survival [4–7]. However, despite the increasing use of these agents, mCRPC remains a lethal disease, mainly due to the emergence of different mechanisms of therapeutic resistance [2], including AR constitutively activation through splice variants, such as splice variant 7 (AR-V7), which is the most common [8], and *AR* gain and/or point mutations [9]. Liquid biopsy represents a useful minimally invasive source of genetic biomarkers [10, 11] thanks to its high concordance between liquid and metastatic tissue biopsies [12], and its potential role in monitoring cancer dynamics and catch tumor heterogeneity [13]. A plethora of studies confirmed that the detection of AR splice variants in circulating tumor cells, whole blood or exosomes and plasma *AR* gene gain are associated with shorter progression-free (PFS) and overall survival (OS) with abiraterone or enzalutamide in mCRPC, even if a direct comparison of these two biomarkers was not provided to date [8, 9, 14–16]. The present trial was aimed at evaluate the impact of *AR* gain and AR-V7 in circulating free DNA and in RNA of plasma-derived exosomes, respectively, on clinical outcome in  chemotherapy-naive mCRPC patients, treated with first-line abiraterone or enzalutamide, in order to identify a biomarker strategy for the early identification of resistance to treatment.

## Materials and methods

This retrospective study evaluated the ability of *AR* gain in circulating free DNA (cfDNA) and AR-V7 in exosomes-derived RNA to predict treatment outcome with first-line abiraterone or enzalutamide. Blood samples for plasma analysis were obtained from eligible patients at baseline and treatment strategy was at the physician’s choice without awareness of AR status. Laboratory investigators were blinded to the clinical information on patients’ outcome.

### Patients enrollment

Patients with mCRPC starting first-line treatment with abiraterone or enzalutamide were enrolled. All patients should have a histologically confirmed diagnosis of prostate adenocarcinoma without neuroendocrine differentiation, ongoing treatment with LHRH analog and no prior treatment with docetaxel. Eligibility criteria included also an Eastern Cooperative Oncology Group (ECOG) performance status (PS) 0–2, adequate cardiac, renal, hepatic, and bone marrow function. Serum prostate-specific antigen (PSA) was assessed within 1 week from starting treatment and monthly thereafter. In addition, biochemical parameters, such as: serum LDH, alkaline phosphatase (ALP), complete blood count were also measured at therapy baseline. Radiographic disease was assessed with computed tomography (CT) and bone scan at the time of screening and every 12 weeks. Progressive disease (PD) was assessed according to Prostate Cancer Clinical Trials Working Group 3 (PCWG3) guidelines [17]. Laboratory analyses were conducted according to the principles set out in the WMA Declaration of Helsinki and the Department of Health and Human Services Belmont Report. All patients provided written informed consent and the study was approved by the IRB of Istituto Scientifico Romagnolo per lo Studio e la Cura dei Tumori (IRST), Meldola, Italy (REC 2192/2013).

### cfDNA and exosome-RNA isolation and analysis

Peripheral blood samples were collected from each patient within 30 days before starting abiraterone or enzalutamide treatment. Overall, 10 ml of blood were collected, transferred in ethylene-diamine-tetra-acetic acid (EDTA) tubes and centrifuged at 1900 × *g* for 10 min at 4 °C within 2 h after drawing. Plasma was divided into 2 aliquots of 2 ml and stored at −80 °C until analysis. Circulating free DNA was extracted with the QIAamp Circulating Nucleic Acid Kit (Qiagen, Valencia, CA) and the total DNA was quantified using the Quant-iT high sensitivity PicoGreen double-stranded DNA Assay Kit (Invitrogen) or by spectrophotometric evaluation (NanoDrop® ND-1000, Milan, Italy). A multiplex digital droplet PCR (ddPCR; Bio-Rad, Hercules, CA) assay was performed to assess plasma AR gain, as previously described [9]. Four reference genes have been used: NSUN3, ElF2C1, AP3B1, and ZXDB at Xp11.21 as a control gene not involving the whole arm of chromosome and each PCR reaction was made with 1–2 ng DNA. *AR* gain was defined as copy number amplification when over the 2.01 threshold [9]. Exosomes-derived RNA extraction was performed using the exoRNeasy kit (Qiagen, Valencia, CA), RNA was transcribed into complementary DNA, amplified using the One-Step RT-ddPCR Kit (Bio-Rad, Hercules, CA) and analyzed by a digital droplet PCR, as previously described [15].

### Statistical analysis

The Kolmogorov–Smirnov test was performed to evaluate the normality of the quantitative data distributions. Kaplan–Meier method was used to evaluate time-to-event outcomes, including clinical and radiographic PFS and OS, survival differences were compared by the log-rank test. To evaluate the correlation between AR-V7 and *AR* gain values, the Spearman’s rho coefficient was calculated. The relationship between AR-V7 status and PSA decline >50% as well as between *AR* gain and PSA decline >50% have been evaluated by Chi-square test. Statistical significance was defined by a *p* value <0.05. All statistical analyses were performed with MedCalc Statistical Software version 14.8.1 (MedCalc Software bvba, Ostend, Belgium). Categorical variables, such as ECOG PS, tumor stage and Gleason score at diagnosis, type of hormonal treatments, presence of bone, lymph node, and visceral metastases, were described by absolute and relative frequencies, whereas quantitative factors as time from diagnosis to start hormonal therapy and baseline total PSA level by median and range.

Neutrophils-to-lymphocytes ratio (NLR) >3 was used to define unfavorable prognosis together with the number of disease sites, considering with a good prognosis patients with one non-visceral metastatic site and with worse prognosis patients with at least two non-visceral metastatic sites (i.e., bone or lymph nodes) or one visceral site [18].

## Results

**Fig. 1:**
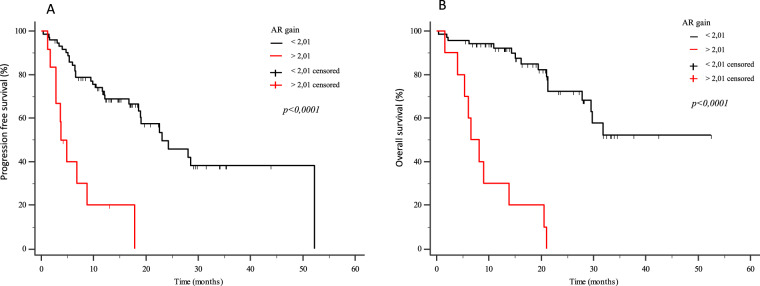
Kaplan–Meier curves. Progression free survival (PFS, **A**) and overall survival (OS, **B**) according to AR-gain status in the overall population.

**Fig. 2:**
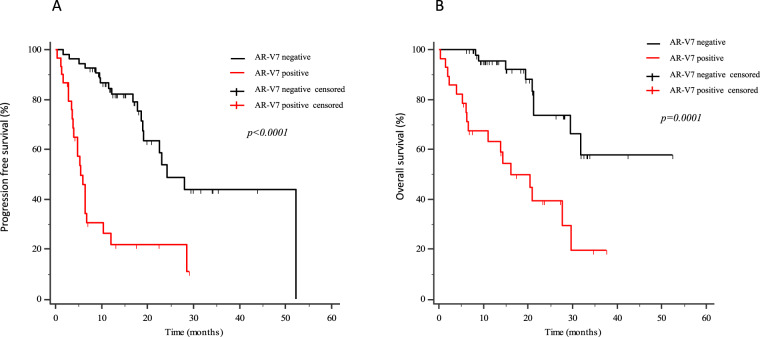
Kaplan–Meier curves. Progression free survival (PFS, **A**) and overall survival (OS, **B**) according to AR-V7 status in the overall population.

**Fig. 3:**
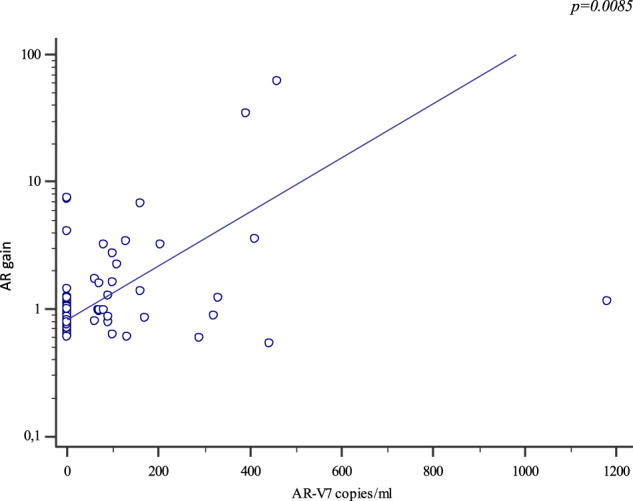
The linear correlation between AR-gain and AR-V7.

**Fig. 4:**
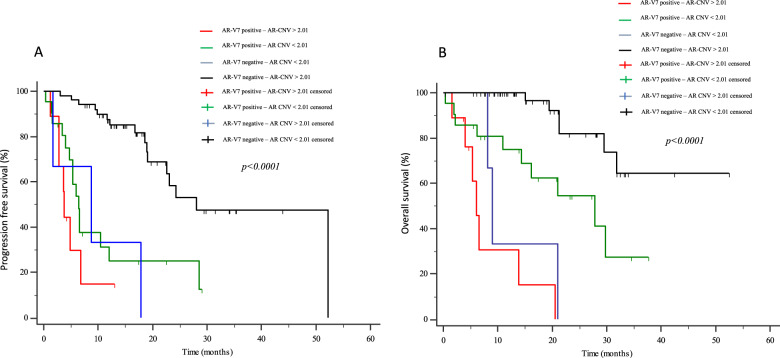
Kaplan–Meier curves. Progression free survival (PFS, **A**) and Overall Survival (OS, **B**) according to AR-gain and AR-V7 combined status in the overall population.

Between December 2014 and October 2018, 84 patients affected by mCRPC were enrolled in this biomarker study with the primary aim of biomarkers evaluation on PFS and OS. Part of these patients (*n* = 16) were previously described in Conteduca et al. [9]. Forty patients were treated with abiraterone and 44 patients received enzalutamide until evidence of PD or life-threatening toxicity. Patients’ median age was 78 years (range 47-91); most patients had an ECOG PS 0–1 (*n* = 77, 84.3%) and showed no visceral metastases (*n* = 79, 94%). Table 1 summarizes patients characteristics. *AR* gain was detected in 14.3% patients and 36% were AR-V7+ at baseline, 9 patients had both *AR* gain and AR-V7. *AR* gain positive patients had a median PFS of 4.8 vs 24.3 months in *AR* normal patients (*p* < 0.0001; Fig. 1A). Median OS was significantly longer in patients with *AR* gain vs *AR* normal (8.17 months vs not reached, *p* < 0.0001; Fig. 1B). Regarding AR-V7 status, median PFS and OS were significantly shorter in AR-V7+ vs AR-V7- patients (PFS: 5.4 vs 24.3 months, *p* < 0.0001; Fig. 2A; OS: 16.2 months vs not reached, *p* = 0.0001; Fig. 2B). Considering both *AR* gain and AR-V7 a significant correlation was observed (*r* = 0.28, *p* = 0.01; Fig. 3), being *AR* gain higher in AR-V7+ patients. The significant difference in terms both of PFS and OS was maintained when patients were stratified as per treatment arm as showed in Supplementary Fig. 1A–D and 2A–D. Stratifying patients accordingly to their AR status, patients without AR variations (no gain and no AR-V7) had longer PFS than patients with *AR* gain only, than patients with AR-V7 only than patients with both *AR* gain and AR-V7 (median PFS 28 vs 8.7 vs 6.4 vs 3.8 months, respectively, *p* < 0.0001; Fig. 4A). Considering the OS, patients with longer survival were still the patients without AR variations, followed by the AR-V7+ only, the *AR* gain only, and *AR* gain and AR-V7+ patients (median OS not reached vs 27.7 vs 8.9 vs 6.1 months, respectively, *p* < 0.0001; Fig. 4B). A significant association between the absence of *AR* gain or AR-V7 and a PSA decline >50% (*p* = 0.0032 and *p* = 0.0013, respectively) was also found. In the univariate and multivariate model, AR-V7 and *AR* gain, resulted significantly associated with shorter PFS and (*p* < 0.0001; Table 2A). However, in the univariate model for the correlation between OS and AR-V7, *AR* gain, NLR, and site score, AR-V7, *AR* gain, NLR were statistically significant (*p* = 0.0003, *p* > 0.0001, *p* = 0.02, respectively), and their association was maintained at the multivariate analysis (*p* = 0.004, *p* < 0.001, *p* = 0.02, respectively; Table 2).

**Table 1:** Patient characteristics.

	Total (*n* = 84)
Age, years Median (range)	78 (47-91)
ECOG PS, *n* (%)	
0–1	77 (84.3)
2	7 (15.7)
Prostatectomy, *n* (%)	
No	41 (48.8)
Yes	43 (51.2)
Radical radiotherapy, *n* (%)	
No	66 (78.6)
Yes	18 (21.4)
Gleason score, *n* (%)	
<8	32 (38.1)
≥8	33 (39.3)
Unknown/missing	19 (22.6)
Bone metastases, *n* (%)	
No	26 (31.0)
Yes	58 (69.0)
Visceral metastases, *n* (%)	
No	79 (94.0)
Yes	5 (6.0)
Nodal metastases, *n* (%)	
No	24 (40.0)
Yes	50 (60.0)
Serum PSA, mg/l	
Median (range)	9.64 (0.2-1555)
Serum LDH, *n* (%)	
<225 U/l	55 (65.5)
≥225^a^ U/l	13 (15.5)
Unknown/missing	16 (19)
Hemoglobin, *n* (%)	
≥12.5^a^ g/l	31 (36.9)
<12.5 g/l	22 (26.2)
Unknown/missing	31 (36.9)
ALP, *n* (%)	
<129 U/l	23 (27.4)
≥129^a^ U/l	6 (7.1)
Unknown/missing	55 (65.5)
NLR, *n* (%)	
<3	35 (41.7)
≥3	18 (21.4)
Unknown/missing	31 (36.9)
SII, *n* (%)	
<535	30 (35.7)
≥535	23 (27.4)
Unknown/missing	31 (36.9)

*ALP* alkaline phosphatase, *ECOG* Eastern Cooperative Oncology Group, *LDH* lactate dehydrogenase, *n* number, *R* neutrophil-to-lymphocyte ratio, *PS* performance status, *PSA* prostate-specific antigen, *SII* systemic immune-inflammation index.

^a^Upper normal value.

**Table 2:** Univariate and multivariate analysis for (A) PFS and (B) OS.

	Univariate		Multivariate	
Variables	HR (95% CI)	*p* value	HR (95% CI)	*p* value
**(A)**				
AR-V7	4.9410 (2.1688–9.3222)	***<0.0001***	2.4227 (1.1646–5.0399)	***0.0185***
AR GAIN	5.5198 (2.575–11.8313)	***<0.0001***	4.6745 (1.9525–11.1914)	***0.0006***
NLR score (>3, <3)	1.7531 (0.9322–3.2969)	0.0831	–	–
Site score	1.6385 (0.88–3.051)	0.1214	–	–
**(B)**				
AR-V7	4.4982 (2.0057–10.0879)	***0.0003***	3.6067 (1.5005–8.6692)	***0.0043***
AR GAIN	12.0667 (4.9479–29.4976)	***<0.0001***	9.3841 (3.4919–25.2188)	***<0.0001***
NLR score (>3, <3)	2.5333 (1.1517–5.5724)	***0.0215***	2.3799 (1.0606–5.3403)	***0.0364***
Site score	2.0801 (0.9551–4.5301)	0.0665	–	

## Discussion

Several treatments are becoming available for mCRPC and biomarkers-driven research is growing, however, there is still no approved biomarker to personalize therapy in clinical practice. AR aberrations, including gain and splice variant 7, represent the most specific and reliable predictive biomarkers to guide treatment decisions in CRPC [8, 9, 16, 19–21]. The present biomarker blinded study enrolled 84 patients undergoing to first-line abiraterone or enzalutamide and collected plasma samples at baseline, demonstrating that the double positivity for *AR* gain and AR-V7 at baseline is associated with shorter PFS and OS. In this study cohort, 14.3% and 36% of men with mCRPC were *AR* gain or AR-V7 positive at baseline on cfDNA and exosomes, respectively, suggesting that AR assessment in liquid biopsy may address up to 50% of AR therapy resistance, also allowing that other mechanisms of resistance to treatment arise. In the present study, a higher proportion of AR-V7 positive cases was identified in first-line compared to previously published papers using CTCs as detection method [20].

This difference may be explained by the limitation of using CTCs as a biomarker. First, many limitations have been reported regarding the use of CTCs due to their fragility, and their morphologic and immune-phenotypic heterogeneity [22]. An indirect comparison of CTCs versus exosomes-derived AR-V7 demonstrates that patients positive for the AR-V7 detected by the two methods have different PFS: 2.2 vs 6.2 mo for CTCs [8] and 3 vs 20 months for exosomes [15] or 5.4 vs 24.3 months (present study). We believe, the shorter PFS of AR-V7- patients in CTCs may be justified by the false negatives in the cohort of patients with AR-V7 analyzed on CTCs, highlighting a higher sensitivity of the exosome approach over CTCs. Moreover, a different expression of AR-V7 in between subgroups of CTCs in CRPC patients has been shown in single cell, confirming high level of molecular heterogeneity [23]. In addition, we observed a significant correlation between AR gain and AR-V7+ (*r* = 0.28, *p* = 0.01; Fig. 3). Recent papers highlighted the association between AR-gain and AR-V7 in both tissue and plasma samples [24, 25]. Interestingly, Kallio et al. showed a correlation between AR gain and AR-splice variants. In mCRPC, the AR co-amplification is the most common molecular alteration and provides a mechanistic explanation for the increase in AR mRNA expression. This increase in AR transcription may be sufficient to produce adequate expression of AR-V7 mRNA to drive resistance to hormonal treatment. Further studies are needed to highlight the biological meaning of this correlation and to understand more of the mechanisms of resistance to treatment.

A recent sub-analysis of the TITAN trial [26] in mCRPC patients treated with apalutamide plus ADT analyzed AR aberrations (AR-V7, *AR* gain, mutations in the AR ligand binding domain) from circulating free DNA and RNA using next-generation sequencing and PCR, respectively. This study provided new insights into how AR aberrations were also associated with a worse outcome in earlier stages of prostate tumor, even at a lower frequency (up to 20%) than that observed in CRPC. Alternative acquired mechanisms of resistance may be AR-dependent or independent [27], and include ligand-binding domain mutations [19], alternative AR splice variants, rearrangements and amplifications [16, 28], glucocorticoid receptor activation [29] and additional compensatory oncogenic pathways [30]. The negative predictive value for PFS of *AR* gain and AR-V7+ using cfDNA and exosomes was independent of clinical prognostic factors such as NLR or site score, supporting the hypothesis that AR status is an independent predictive biomarker of resistance to abiraterone and enzalutamide. Considering the OS, in addition to *AR* gain and AR-V7+ also the NRL showed to be an independent predictive factor, suggesting that, being NLR the link between innate (neutrophil) and adaptive (lymphocyte) immune response, NLR may represent the dynamic change of host in the inflammatory and immune response, and treatment effect [18].

The small sample size of the cohort and its retrospective design represent the major limitations of the present study. Other AR aberrations such as mutations [9] and somatic or germ line aberrations not involving AR such as genomic alterations in RB1, TP53, PTEN, homologous recombination repair were not included in the analysis [31]. Moreover, in the present paper the cut off of 2.01 for AR gain has been used, accordingly to previously published data by our group [9], even if it is known that  cut offs for AR gain vary among the detection methods and this could be a bias in order to use this test across different laboratories [32, 33]. Methods used to assess AR status did not permit to analyze tumor content burden, often providing useful information regarding prediction to therapeutic response and prognosis, mainly in advanced prostate cancer. Nevertheless, our results suggest that a prospective larger trial assessing overall circulating AR status is warranted. Particularly, a randomization between AR targeting agents and chemotherapy could be challenging thanks to patients’ stratification by plasma AR status, considering that detection of plasma AR gain and AR splice variants in CTCs [34, 35] was also performed separately in mCRPC patients treated with taxanes.

We found that the presence of AR-V7 or/and AR gain at baseline were related with more aggressive cancers and suggest these should be considered when deciding on treatment. In conclusion, the present study demonstrates that the AR gain/AR-V7 combined analysis has a strong value as prognostic and predictive biomarker, since both OS and PFS were significantly shorter compared to patients without any AR aberration (PFS 3.8 vs 28 month, respectively; OS 6.1 vs not reached, respectively; *p* < 0.0001).

Indeed, analysis of AR gain in circulating tumor DNA and AR-V7 in exosomes, in conjunction with standard clinical assessment, may help identifying men who will not benefit from abiraterone or enzalutamide therapy. Testing AR status by liquid biopsy (cell free DNA and exosomes) already demonstrated to be affordable and that can be widely implemented in clinical laboratories [9, 15, 16, 20, 36]. Moreover, the standardization and the clinical validation of a liquid biopsy-based assay able to detect predictive biomarkers of resistance is a critical point, in order to develop a precision medicine algorithm for mCRPC patients.

## Supplementary information

Supplementary figures legend

Supplemental Material 1

Supplemental Material 2

## Data Availability

The dataset used in the current study is available as unpublished material if requested.
