# Supplementary material for: Androgen receptor gain in circulating free DNA and splicing variant 7 in exosomes predict clinical outcome in CRPC patients treated with abiraterone and enzalutamide
Source: Prostate Cancer Prostatic Dis. 2021 Jan 26;24(2):524–31. doi: 10.1038/s41391-020-00309-w (PMC8134038; doi:10.1038/s41391-020-00309-w)
Supplement: Supplementary file 1 — Supplementary figures legend [file 41391_2020_309_MOESM1_ESM.docx]

**Supplementary figures legend**

**Supplementary figure 1.** PFS (A) and OS (B) according to AR gain status in the abiraterone treated population. PFS (C) and OS (D) according to AR gain status in the enzalutamide treated population.

**Supplementary figure 2.** PFS (A) and OS (B) according to AR-V7 status in the abiraterone treated population. PFS (C) and OS (D) according to AR-V7 status in the enzalutamide treated population
