# Supplementary material for: Androgen receptor gain in circulating free DNA and splicing variant 7 in exosomes predict clinical outcome in CRPC patients treated with abiraterone and enzalutamide
Source: Prostate Cancer Prostatic Dis. 2021 Jan 26;24(2):524–31. doi: 10.1038/s41391-020-00309-w (PMC8134038; doi:10.1038/s41391-020-00309-w)

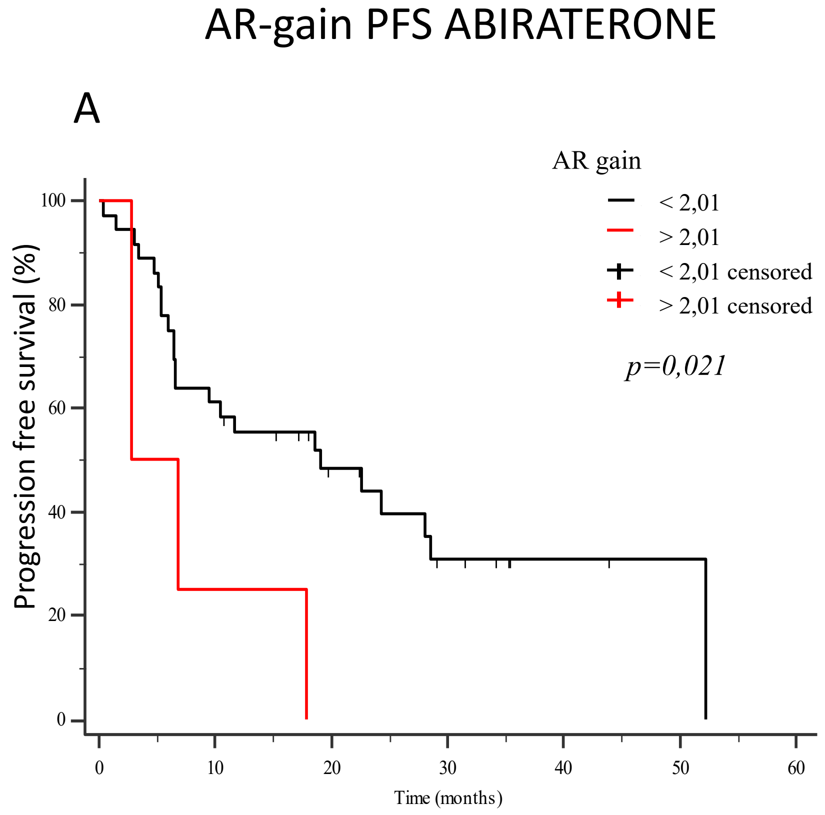
AR-gain PFS ABIRATERONE
A
AR gain
< 2,01
> 2,01
< 2,01 censored
> 2,01 censored
p=0,021
Progression free survival (%)
Time (months)

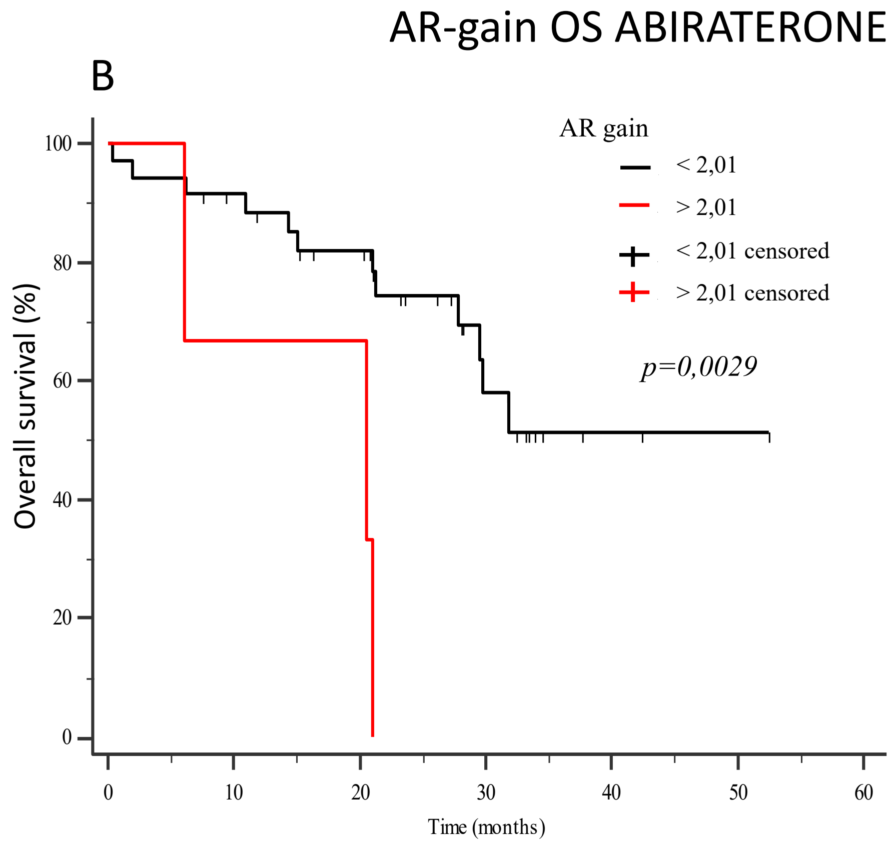
AR-gain OS ABIRATERONE
B
AR gain
< 2,01
> 2,01
< 2,01 censored
> 2,01 censored
p=0,0029
Overall survival (%)
Time (months)

AR-gain PFS ENZALUTAMIDE

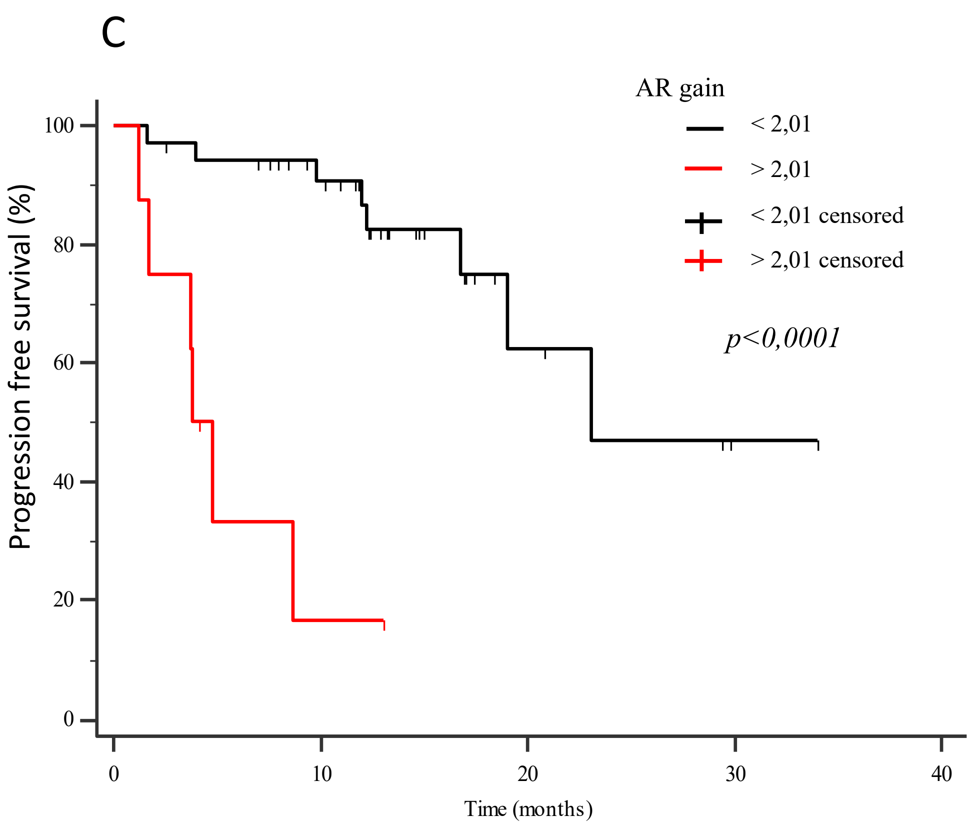

AR-gain OS ENZALUTAMIDE

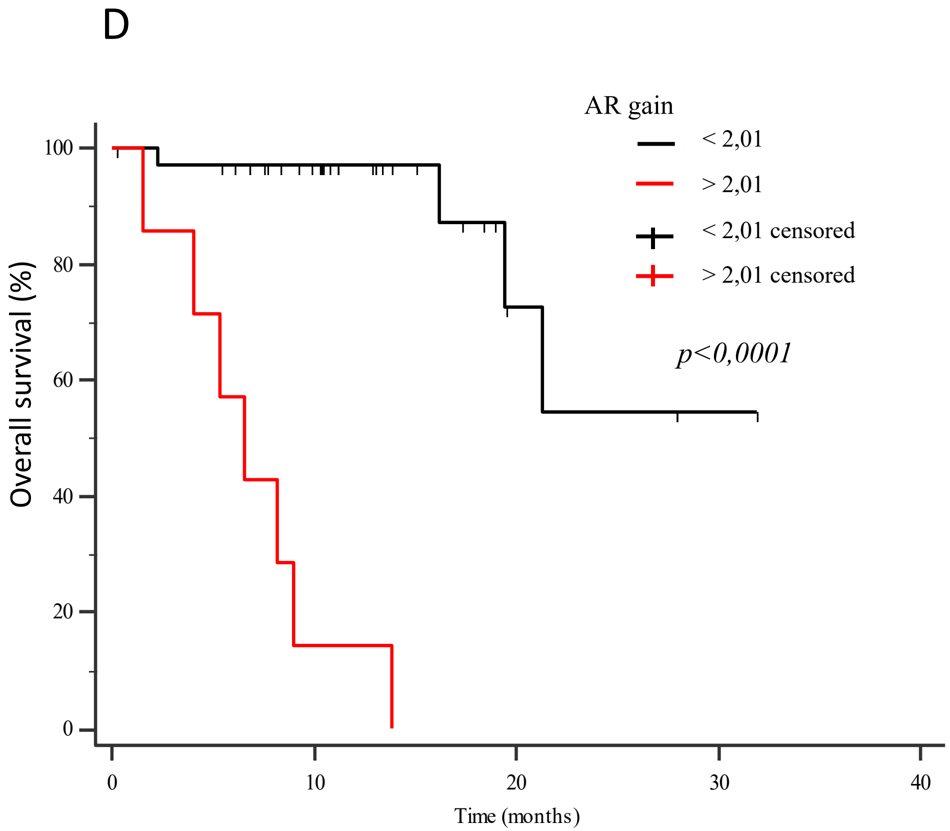

Supplement: Supplementary file 2 — Supplemental Material 1 [file 41391_2020_309_MOESM2_ESM.pdf]
