# Supplementary figures and images for: Androgen receptor gain in circulating free DNA and splicing variant 7 in exosomes predict clinical outcome in CRPC patients treated with abiraterone and enzalutamide
Source: Prostate Cancer Prostatic Dis. 2021 Jan 26;24(2):524–31. doi: 10.1038/s41391-020-00309-w (PMC8134038; doi:10.1038/s41391-020-00309-w)

**A** AR-V7 PFS ABIRATERONE

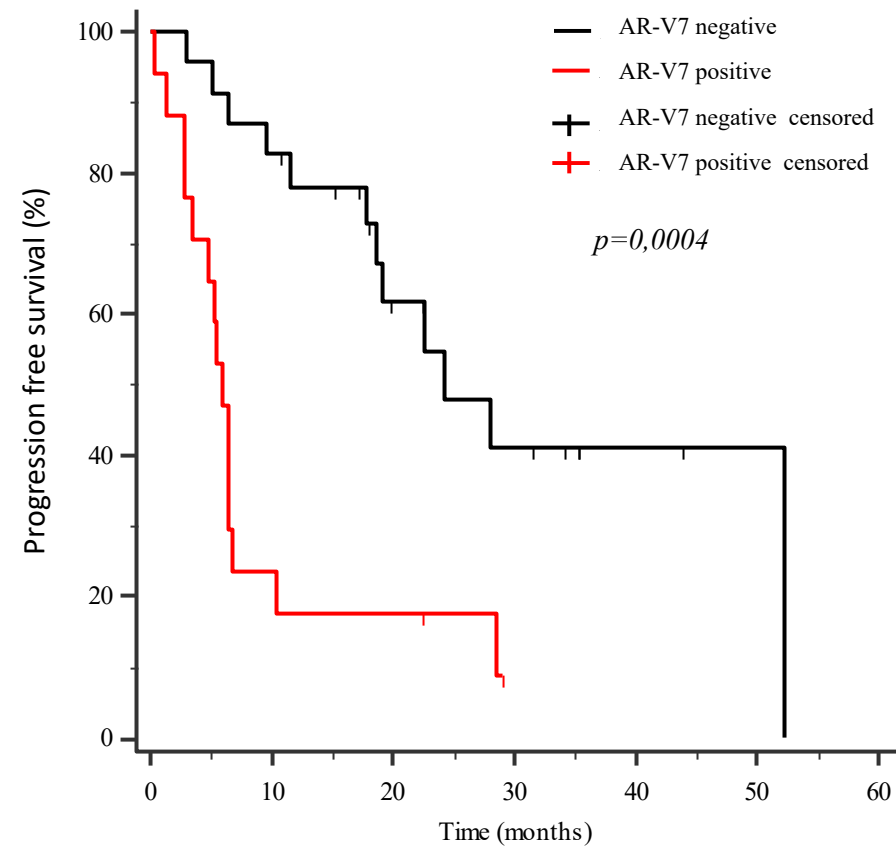

**B** AR-V7 OS ABIRATERONE

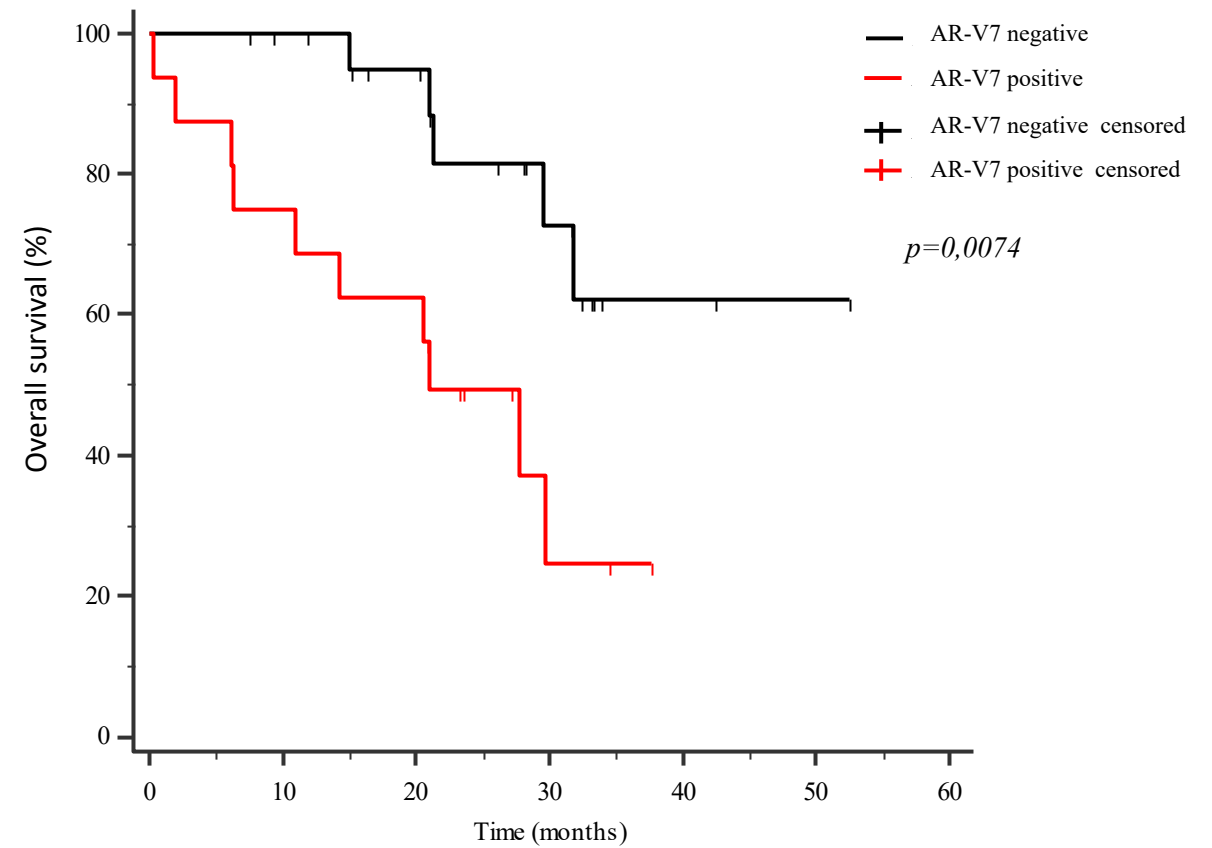

## AR-V7 PFS ENZALUTAMIDE

A

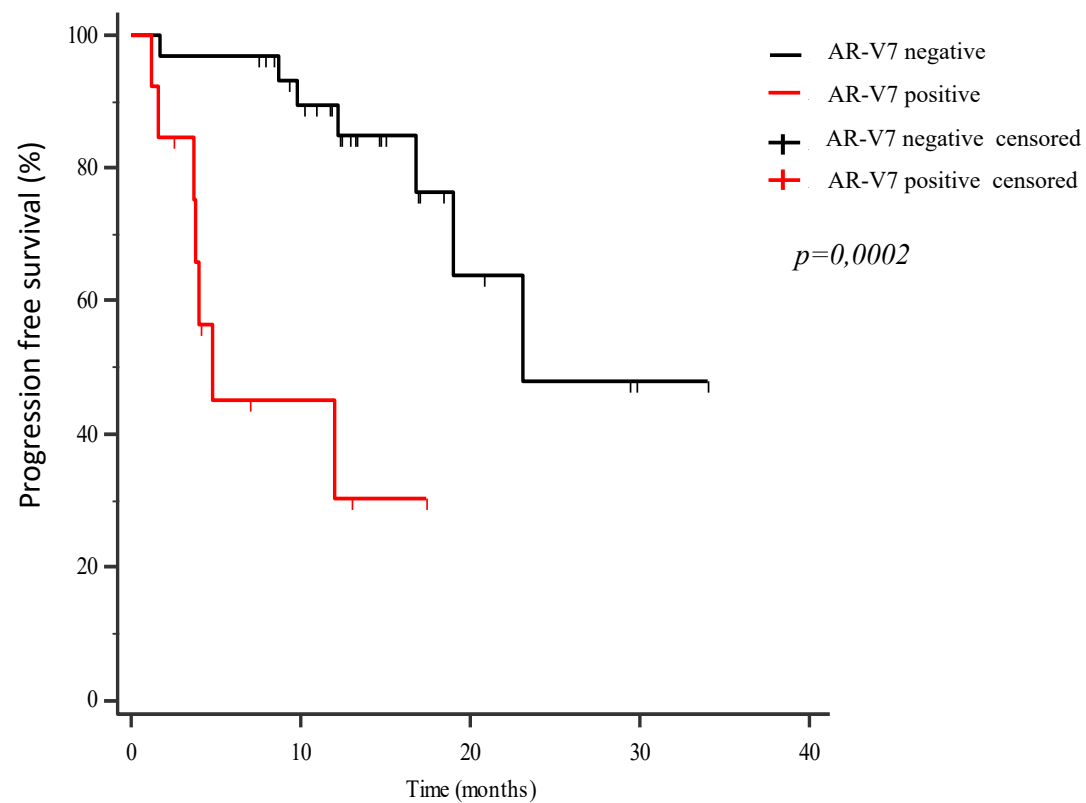

## AR-V7 OS ENZALUTAMIDE

B

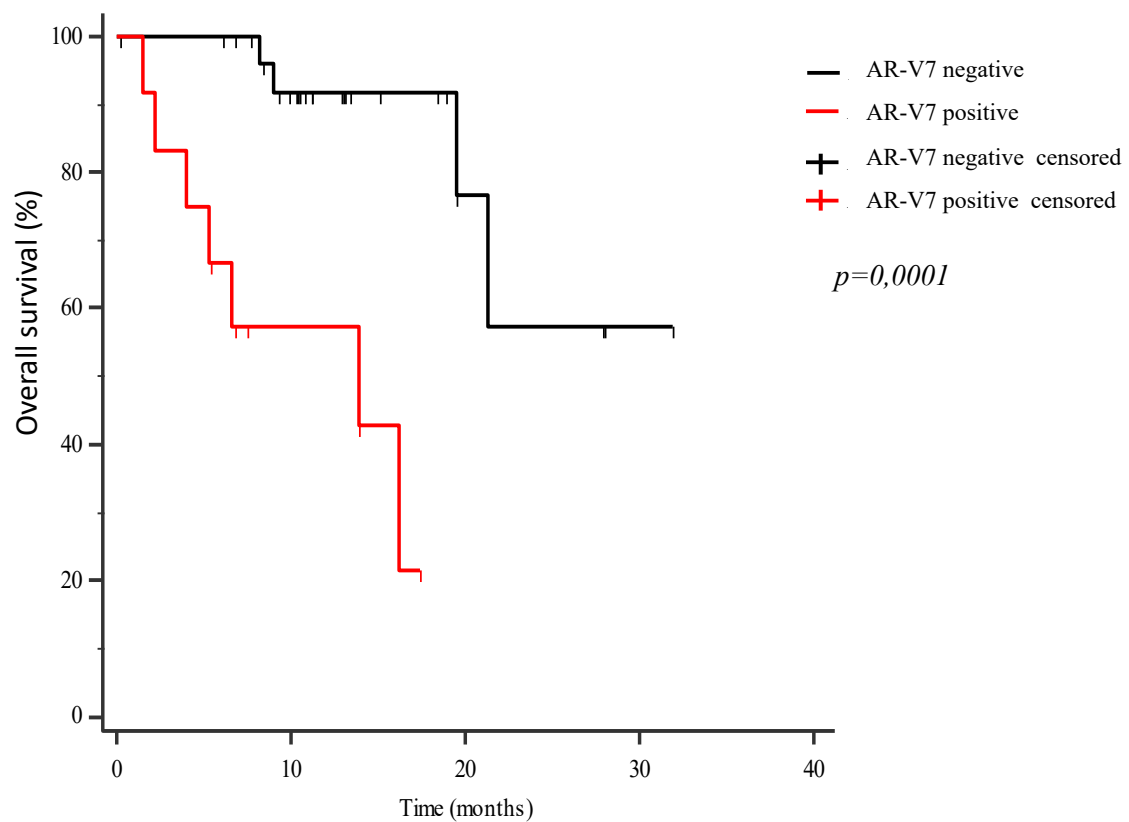

Supplement: Supplementary file 3 — Supplemental Material 2 [file 41391_2020_309_MOESM3_ESM.pdf]
